# Primary Small Bowel Volvulus: An Unusual Cause of Small Bowel Obstruction

**DOI:** 10.7759/cureus.6465

**Published:** 2019-12-25

**Authors:** Chi Lap Nicholas Tsang, Christo T Joseph, Marie Shella B De Robles, Soni Putnis

**Affiliations:** 1 Surgery, The Wollongong Hospital, Wollongong, AUS; 2 General Surgery, The Wollongong Hospital, Wollongong, AUS; 3 Surgery, Philippine General Hospital, Manila, PHL; 4 Colorectal Surgery, The Wollongong Hospital, Wollongong, AUS

**Keywords:** small bowel, volvulus, mesenteric, midgut, obstruction

## Abstract

We present the case of a 78-year-old female who presented to the emergency department with small bowel obstruction in a virgin abdomen. Although the patient did not have peritonism and biochemical investigations did not reveal alarming features of ischemia, an abdominal computed tomography (CT) scan was suggestive of small bowel volvulus (SBV), and operative exploration was pursued. No obvious cause was identified aside from hard stools throughout the colon and a diagnosis of primary SBV was determined. She was subsequently discharged symptom-free on day seven post-operatively. She re-presented on day 10 post-operatively with a similar history, examination, and abdominal CT findings suggestive of SBV recurrence. Her volvulus slowly resolved post administration of rectal enemas and did not require any further operative intervention; she was discharged on day eight of re-admission (day 19 post-operatively) with no recurrence of her symptoms on a regular diet. In this article, we discuss the management of SBV.

## Introduction

Small bowel volvulus (SBV) is a rare surgical pathology with only 1% of small bowel obstruction being attributed to volvulus [1]. It occurs as a result of rotation of the gut lumen along an axis or mesenteric twisting causing obstruction [2]. Life-threatening sequelae of SBV can include but is not exclusive of ischemia, necrosis, and perforation [2-3]. Management can consist of conservative medical and/or surgical measures including bowel resection depending on the clinical picture of the patient and surgical pathology encountered intraoperatively [2,4]. In this case report, we discuss a rare case of primary SBV as well as a review of literature.

## Case presentation

**Figure 1:**
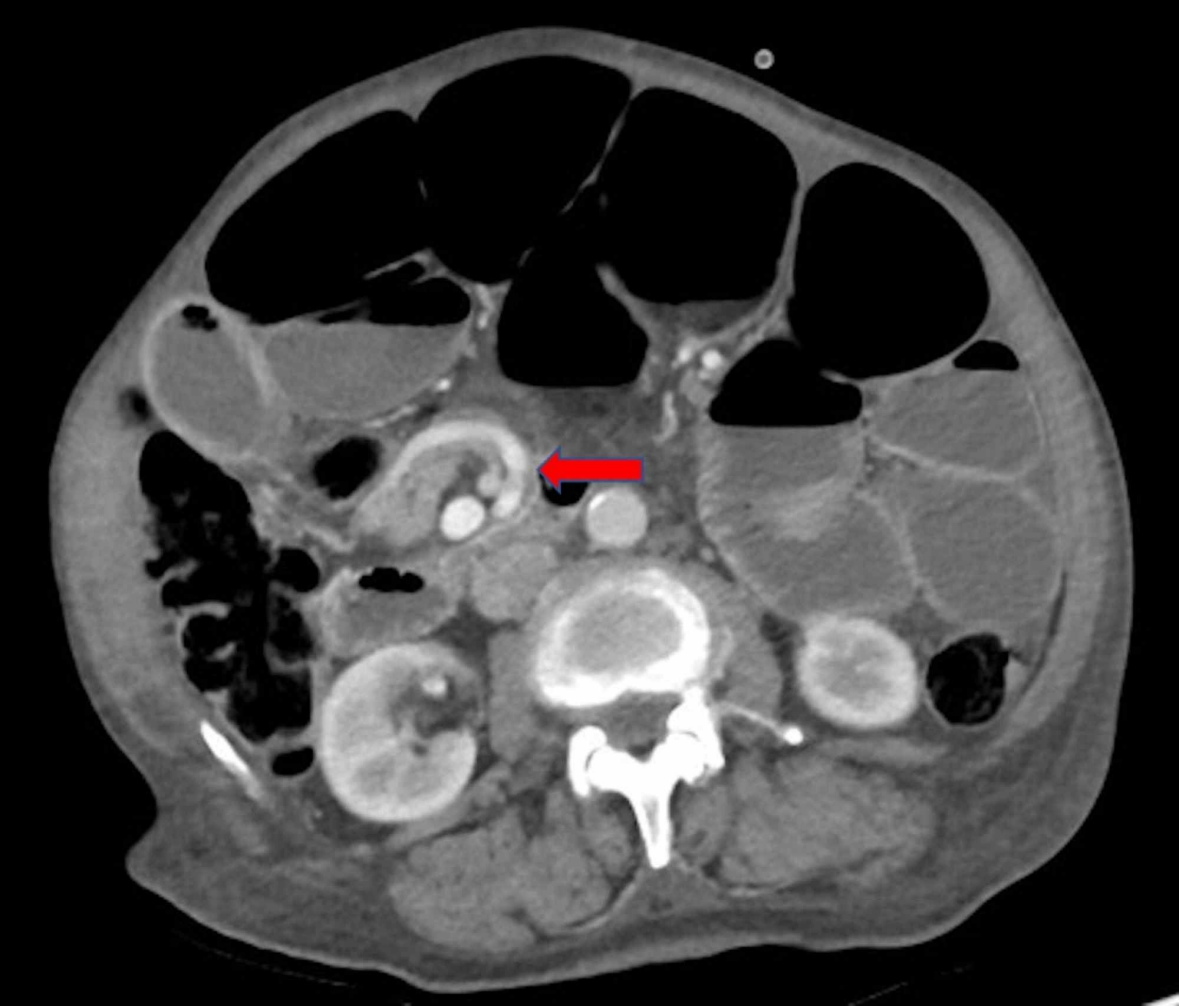
Axial slice of abdominal computed tomography (CT) after contrast media administration, in portal phase demonstrating “whirlpool sign” of mesenteric vessels, suggestive of small bowel volvulus (arrow) with dilated loops of small bowel

**Figure 2:**
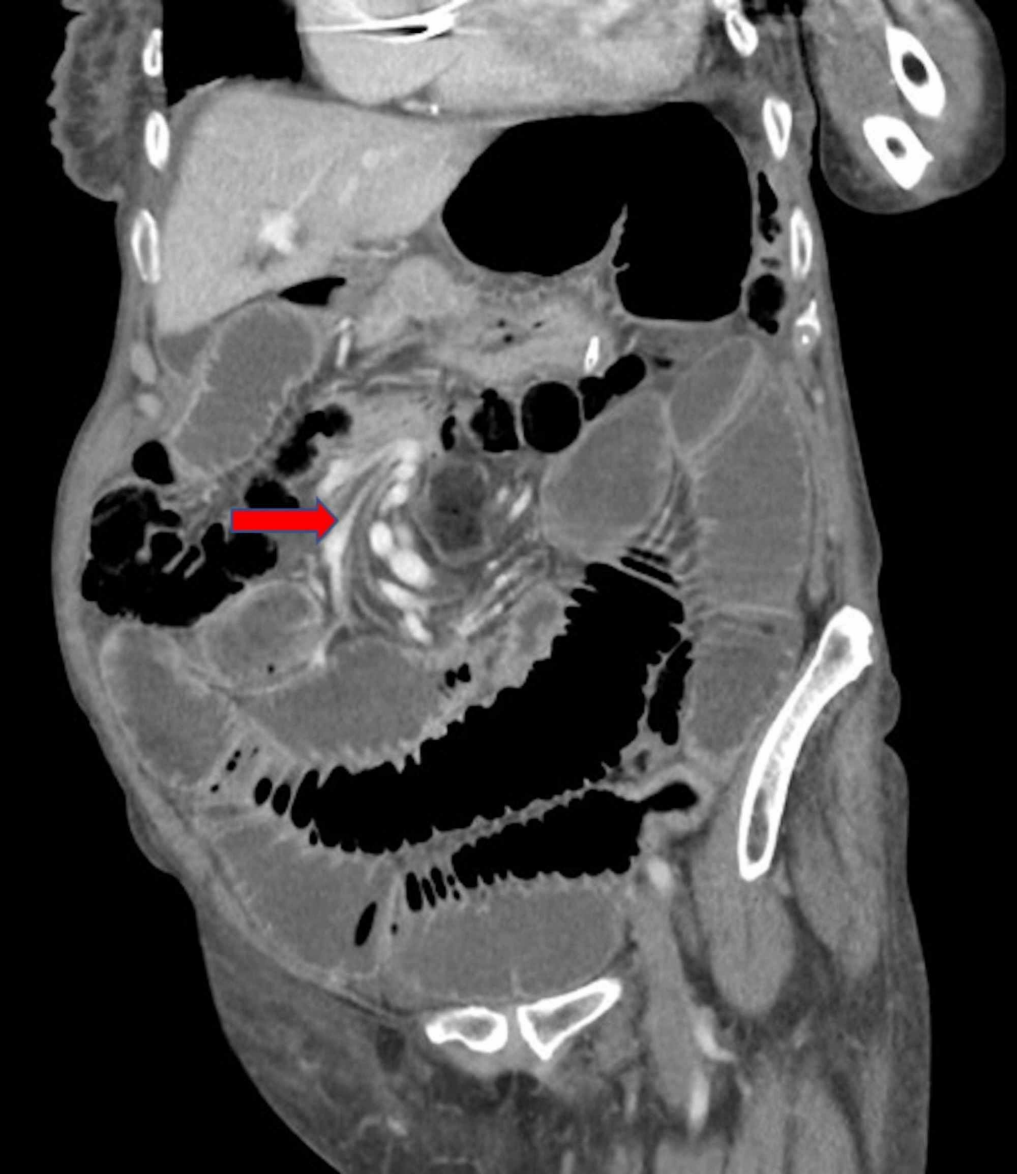
Coronal slice of abdominal computed tomography (CT) after contrast media administration, in portal phase demonstrating “whirlpool sign” of mesenteric vessels, suggestive of small bowel volvulus (arrow) with dilated loops of small bowel

A 78-year-old female of Macedonian descent presented to the emergency department with a one-day history of abdominal distention and nonspecific intermittent central abdominal discomfort with an isolated episode of dark vomit whilst in the emergency department. Her medical background included paroxysmal atrial fibrillation with a recent pacemaker insertion for bradycardia, hypertension, gastroesophageal reflux disease, glaucoma, left-sided cerebrovascular event, pulmonary hypertension, and iron deficiency anaemia requiring iron transfusions with normal renal function. She had no previous abdominal surgical history and had a normal gastroscopy and colonoscopy four years ago. On further history, she suffered from chronic constipation. Examination revealed a haemodynamically stable patient with a grossly distended abdomen and palpable small bowel loops with no peritonism. Rectal examination revealed scant amounts of hard faeces palpable in the rectal vault but was otherwise unremarkable. Biochemical analysis was unremarkable with normal white cells, C-reactive protein (CRP), and an International Normalized Ratio (INR) of 1.9 on warfarin. Abdominal computed tomography (CT) was conducted demonstrating small bowel obstruction with associated a “whirlpool sign” of SBV (Figures 1-2).

**Figure 3:**
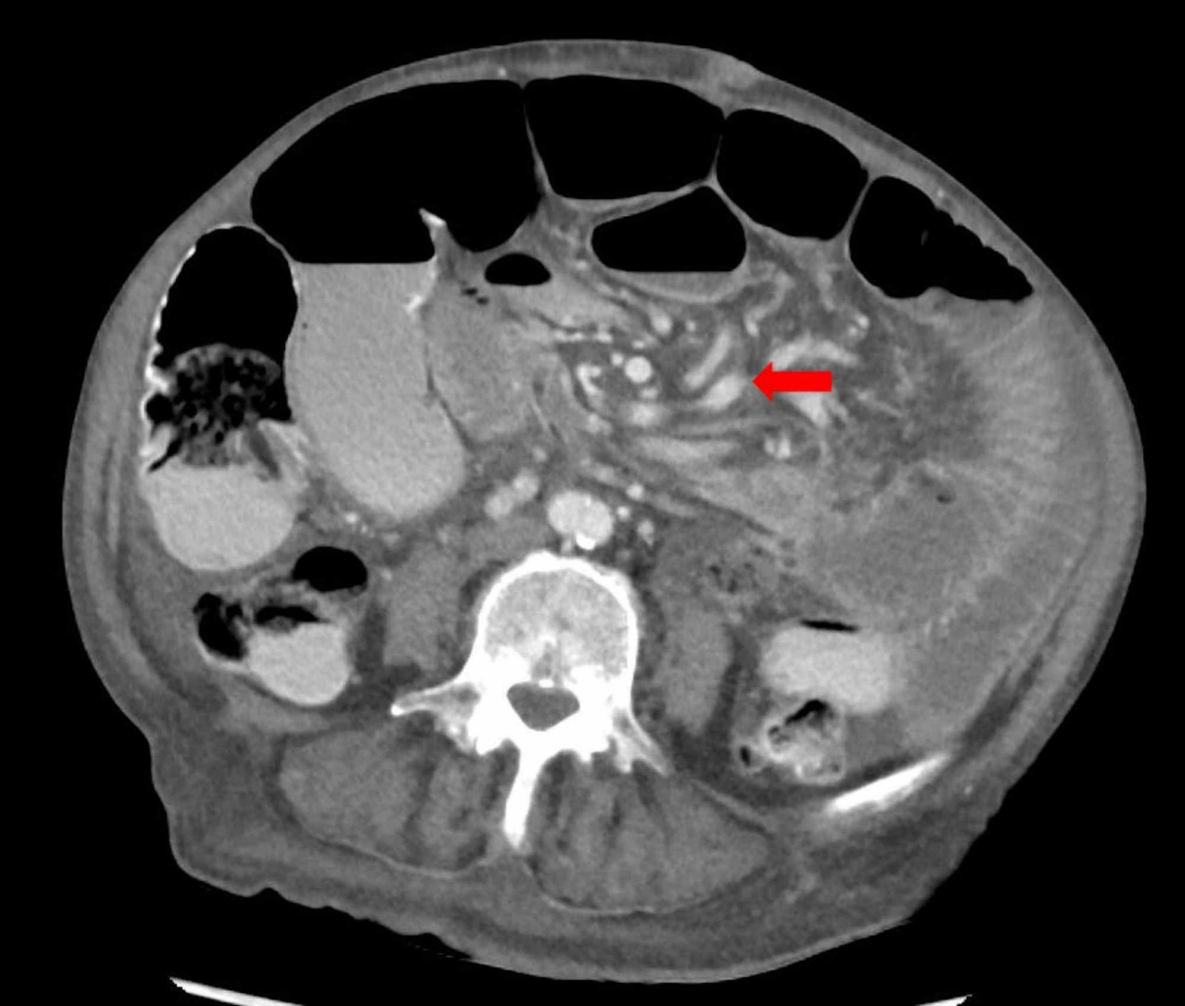
Post-operative axial slice of abdominal computed tomography (CT) in portal phase demonstrating recurrence of “whirlpool sign” of mesenteric vessels, suggestive of small bowel volvulus (arrow) with dilated loops of small bowel

A nasogastric tube (NGT) was inserted with drainage of bilious contents and mild relief of distention. An indwelling catheter (IDC) was inserted for fluid balance and fluid resuscitation was commenced. Warfarin was reversed with prothrombinex and operative exploration was pursued to exclude abdominal catastrophe. A laparoscopic approach was not feasible due to marked distention in a very thin individual and so a decision was made for exploratory laparotomy. Intraoperative findings demonstrated marked small bowel distention with mesenteric twisting in the right abdomen involving the ileum, with collapsed colon distally and hard stools palpable within the colon. No adhesions or congenital bands were noted that would have accounted for the volvulus and a complete small bowel run showed healthy viable bowel from the duodenal-jejunal flexure to terminal ileum. A simple devolvulation was performed and approximately one litre of small bowel content was milked retrograde into the stomach and drained via the NGT. The abdomen was closed thereafter with 1-0 polydioxanone suture (PDS) to fascia and 3-0 monocryl to skin. Her admission was complicated with a line-associated deep vein thrombosis despite supratherapeutic INR levels and had transient recurrence of her abdominal distention resolving with regular twice daily rectal enemas and temporary reinsertion of NGT. She was discharged on day seven post-operatively symptom free and with opening bowels. She re-presented with a similar history, clinical signs, and abdominal CT scan on day 11 post-operatively (as seen in Figure 3) which was treated successfully with regular rectal enemas and insertion of NGT; she was discharged symptom-free with a non-distended abdomen on day eight of re-admission (day 19 post-operatively).

## Discussion

SBV is an uncommon cause of small bowel obstruction [1]. Separated into primary and secondary causes, primary SBV is defined as SBV with no pre-existing anatomical abnormalities; whereas, secondary SBV occurs as a result of a myriad of anatomical pathologies including congenital abnormalities, bands, adhesions, and tumours [2]. Li et al., in their retrospective review of 31 patients, found no obvious cause of primary SBV and alluded to the difficulty in its diagnosis and management, although other retrospective studies have described a slight female predilection [1-2]. 

Workup of SBV should include a thorough history and physical examination, biochemical analysis, and radiographic investigation ideally with an abdominal CT looking for a “whirlpool” or “barber pole” sign of mesenteric volvulus [5]. Among other biochemical markers, an elevated lactate level can signal mesenteric ischemia or necrosis, although this too can be normal despite abdominal catastrophe [6].

In the case of our patient, it is theorised that constipation with hard stools in the colon was a key factor in her development of recurrent mesenteric volvulus. This was demonstrated by the resolution of her abdominal distention with aperient use on multiple occasions throughout her initial and re-admission. Although constipation has been identified as a risk factor for the development of colonic volvulus, there is a paucity of literature regarding its contribution to the development of SBV [7]. Nonetheless, the best support to our claim lies in the fact that this patient did not receive any rectal aperients in between her admissions for SBV and distention resolved with administration of rectal enemas. It is unknown whether this patient’s recent pacemaker insertion contributed to the development of SBV, however, fasting followed by a large meal has been attributed to volvulus in Muslims during Ramadan and a similar scenario may have occurred when this patient had to fast for a procedure [4].

The general management of SBV consists of surgical exploration as well as supportive management with the insertion of NGT, IDC, strict fluid balance, venous thromboembolism prophylaxis, and intensive care involvement as necessary [3]. Operative investigation is recommended in accordance with international guidelines such as the World Society of Emergency Surgery to exclude mesenteric ischemia, necrosis, and perforation [8]. Although laparotomy was the traditional method of exploration, laparoscopic surgery has also been employed [2]. In regards to surgical methods to prevent further volvulus in primary SBV with viable bowel, there is controversy regarding whether simple devolvulation or further measures such as intestinal fixation or prophylactic bowel resection provides the best long term solution [2]. There is a paucity of data to strongly support any of these methods as the gold standard surgical management.

In our case, the patient underwent a simple devolvulation with recurrence of her volvulus post discharge and concurrent aperients cessation. It is difficult to determine whether other surgical methods such as intestinal fixation would have been beneficial for this patient but as her symptoms were reversible with the use of rectal enemas; further surgery was not pursued.

## Conclusions

SBV is an uncommon but potentially serious condition causing small bowel obstruction. Clinicians must be aware of this possibility in their differential especially in patients with a virgin abdomen. Surgeons should have a low threshold for operative investigation although further research is required to ascertain the ideal surgical management method.
